# Soft Polymeric Matrix-Mediated Stabilization of Bulk Heterojunction Morphology for Thermally Robust Organic Photovoltaics

**DOI:** 10.3390/gels12080750

**Published:** 2026-08-21

**Authors:** Unyong Lee, Junpyo Seo, Minwoo Nam

**Affiliations:** Department of Electronic Engineering, Keimyung University, Daegu 42601, Republic of Korea

**Keywords:** organic photovoltaics, bulk heterojunction, thermoplastic elastomer, soft polymeric matrix, gel-related materials, morphology stabilization, thermal stability

## Abstract

Suppressing thermally driven morphological evolution while preserving efficient charge transport pathways remains a critical challenge for improving the long-term stability of organic photovoltaics (OPVs). Herein, a soft polymeric matrix strategy based on gel-related soft material concepts is demonstrated for stabilizing bulk heterojunction (BHJ) morphology and simultaneously improving the efficiency and thermal durability of OPVs. The incorporation of an optimal 5 wt% polystyrene-*block*-poly(ethylene-*ran*-butylene)-*block*-polystyrene (SEBS) as a soft polymeric matrix component into a PM6:Y6 blend modulates the nanoscale morphology and local packing characteristics of the acceptor phase. These changes improve charge-transport balance and charge collection, increasing the power conversion efficiency (PCE) from 14.27% to 15.22%, corresponding to a 6.7% relative enhancement over the control device. More importantly, after 10 days of thermal aging at 85 °C, the SEBS device retains 87.1% of its initial PCE, compared with 72.5% for the control device. Complementary morphological and spectroscopic analyses reveal suppressed thermally induced structural evolution and aggregation in the SEBS-containing films. These findings demonstrate that a gel-related soft polymeric matrix can regulate BHJ organization and mitigate thermally driven morphological evolution, providing a simple strategy for addressing the efficiency–stability trade-off and realizing thermally robust OPVs.

## 1. Introduction

Organic photovoltaics (OPVs) have attracted considerable attention as next-generation solar energy technologies owing to their mechanical flexibility, optical semitransparency, and compatibility with low-temperature solution processing [1,2]. In high-performance OPVs, the bulk heterojunction (BHJ) morphology plays a decisive role in determining photovoltaic operation because efficient charge generation and extraction depend on the formation of sufficient donor–acceptor interfaces together with continuous pathways for charge transport. Favorable BHJ structures are generally established through kinetically controlled phase separation during solution processing. In addition to material properties and composition, the deposition technique also influences film formation and morphology, thereby affecting photovoltaic performance [3,4,5]. However, the resulting BHJ morphologies are often thermodynamically metastable and gradually relax during device operation or storage [6,7]. Under prolonged thermal stress, molecular rearrangement and further phase separation cause excessive aggregation and domain coarsening, thereby reducing the donor–acceptor interfacial area, disrupting percolated charge transport networks, and ultimately degrading the photovoltaic performance [7,8,9]. Therefore, suppressing thermally driven morphological evolution while preserving the as-formed charge transport pathways remains an important challenge in overcoming the efficiency–stability bottleneck of OPVs. This challenge motivates the design of soft polymeric matrix environments that suppress undesirable molecular diffusion and structural relaxation while preserving photogenerated charge transport.

Considerable efforts have been devoted to stabilizing BHJ morphologies without sacrificing the photovoltaic efficiency. Among the various strategies, the incorporation of a third solid component into BHJ active layers has emerged as an effective approach for regulating film formation and suppressing unfavorable morphological evolution [10,11]. Third components contribute to BHJ stabilization through distinct mechanisms. Nonvolatile or volatilizable solid additives serve as alternatives to conventional high-boiling-point solvent additives by enabling more controlled aggregation and drying kinetics, thereby promoting favorable donor–acceptor organization while minimizing the instability associated with residual solvent additives [12,13]. Polymeric or polymer acceptor additives restrict excessive molecular diffusion and thermally driven phase separation, thereby helping to preserve percolated charge transport pathways during accelerated aging [14,15]. Cross-linkable third components immobilize an optimized BHJ structure by forming covalently bonded or physically robust networks, resulting in the improved thermal durability of OPVs [16,17]. Although these approaches demonstrate the versatility of third-component engineering, some require electronically active conjugated materials, deliberately designed intermolecular interactions, or additional cross-linking procedures, thereby increasing material and processing complexity and potentially disrupting the delicate balance between phase separation and charge transport in the BHJ active layer. Accordingly, an electrically inactive soft polymeric matrix capable of regulating molecular mobility and structural relaxation offers an attractive route for stabilizing BHJ morphology without imposing substantial electronic perturbation on the donor–acceptor network. This matrix-stabilization concept is closely aligned with gel-related soft material design principles, in which compliant polymeric environments are used to regulate molecular mobility, morphology, mechanical compliance, and long-term structural stability [18,19]. In this context, applying gel-related matrix concepts to OPV active layers provides a rational strategy for stabilizing non-gel functional energy-conversion materials under thermal stress.

Insulating polymers and thermoplastic elastomers represent a distinct class of third components because they modify the soft polymeric environment during BHJ formation without directly participating in photoinduced charge generation [20,21,22]. Polystyrene-*block*-poly(ethylene-*ran*-butylene)-*block*-polystyrene (SEBS) is a commercially available insulating thermoplastic elastomer that combines low cost with high ductility, mechanical softness, and facile processability [23,24]. The compliant elastomeric character and matrix-stabilizing role of SEBS make it a relevant soft polymeric component for implementing gel-related matrix-stabilization concepts in non-gel functional energy materials. SEBS and related elastomers have primarily been investigated as mechanical toughening components in OPV active layers or as interfacial bonding layers in flexible devices, where they increase the crack-onset strain, reduce film stiffness, enhance fracture toughness, and strengthen the adhesion between functional layers [24,25,26,27]. These studies have established the utility of SEBS for improving the mechanical robustness of flexible OPVs. In addition to providing mechanical reinforcement, SEBS influences the morphology and molecular packing of organic semiconductor blends as a physically compliant polymeric component. At appropriately controlled loadings, SEBS incorporation promotes favorable molecular packing, improves charge carrier mobility, and regulates donor–acceptor domain organization [24,25,26]. Despite the growing recognition of its influence on the initial BHJ morphology, considerably less attention has been paid to its role in the long-term retention of BHJ nanostructures under operational conditions. This issue is particularly important because molecular diffusion and phase evolution continue during thermal aging, gradually altering the domain dimensions, interfacial configuration, and charge transport pathways [7,28,29]. Therefore, it is necessary to clarify whether and how SEBS-induced changes in the initial BHJ morphology are related to its subsequent structural evolution under prolonged thermal stress.

Here, we demonstrate soft polymeric matrix-mediated stabilization of BHJ morphology by incorporating a small amount of SEBS into a non-fullerene acceptor-based active blend. The incorporation of SEBS modified the nanoscale organization and aggregation characteristics of the PM6:Y6 photoactive blend without inducing detrimental macroscopic phase separation. These morphological changes were accompanied by enhanced charge carrier mobilities, more balanced hole and electron transport, and improved charge collection, resulting in enhanced photovoltaic performance compared with that of the control devices. More importantly, the SEBS-containing devices exhibited reduced performance degradation during prolonged thermal aging and suppressed morphological changes associated with excessive aggregation and domain coarsening. The combined experimental results suggest that the SEBS-mediated regulation of the initial BHJ structure is closely associated with improved morphological retention under thermal stress. These findings establish SEBS as a compliant soft polymeric matrix component that stabilizes non-gel functional BHJ active layers and demonstrate the potential of gel-related soft material concepts for overcoming the efficiency–stability bottleneck in thermally robust OPVs.

## 2. Results and Discussion

**Figure 1:**
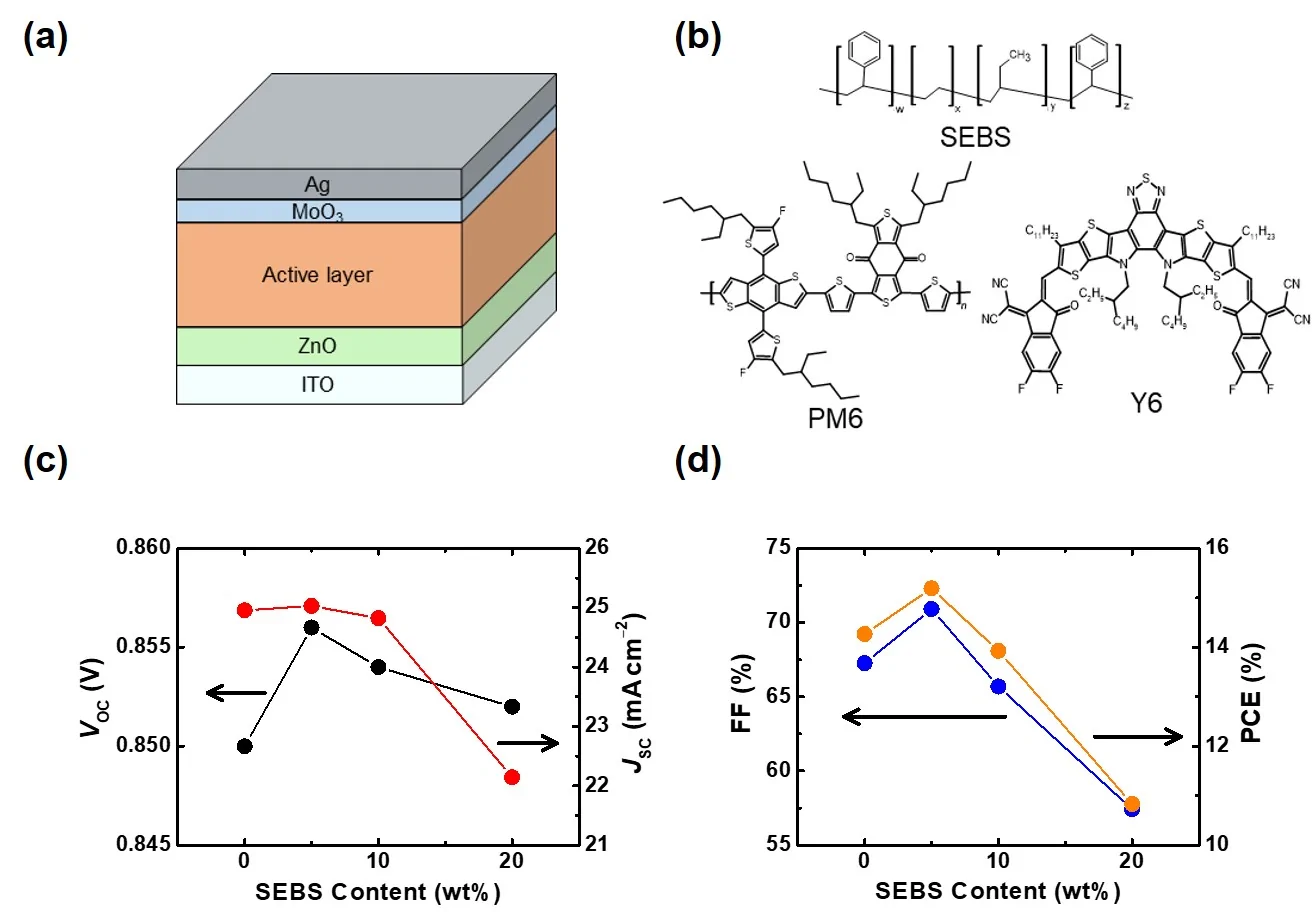
(**a**) Schematic illustration of the inverted OPV device architecture and (**b**) chemical structures of the materials used in the active layer. Photovoltaic parameters of the OPVs as a function of the SEBS content measured under AM 1.5G illumination at 100 mW cm^−2^: (**c**) *V*_OC_ and *J*_SC_ and (**d**) FF and PCE.

Figure 1a shows a schematic diagram of the inverted OPV device architecture. The device consists of sequential layers of indium tin oxide (ITO)/ZnO/active layer/MoO_3_/Ag. Figure 1b presents the molecular structures of the materials used in the active layer. A PM6:Y6 blend, widely recognized as a high-efficiency BHJ system, was employed as the active layer [30]. To systematically investigate the effect of SEBS incorporation, devices were fabricated with varying SEBS content in the BHJ active layer. SEBS is an electrically insulating thermoplastic elastomer that functions as a physically compliant soft polymeric matrix component within the blend. Its elastomeric softness and matrix-stabilizing characteristics make it a useful model component for examining how gel-related soft material concepts can be extended to BHJ active layers. Therefore, unlike electronically active third components, SEBS is expected to influence phase separation behavior, nanoscale morphology, and charge transport pathways mainly through matrix-mediated physical regulation rather than direct participation in photoinduced charge generation [24,25,26]. Figure 1c,d show the variations in photovoltaic parameters as a function of the SEBS content. The short-circuit current density (*J*_SC_) and fill factor (FF) exhibited a noticeable dependence on the SEBS content, whereas the effect on the open-circuit voltage (*V*_OC_) was relatively small. Upon the introduction of a small amount of SEBS (5 wt%), a noticeable increase in the FF was observed, accompanied by slight increases in the *J*_SC_ and *V*_OC_. Consequently, the power conversion efficiency (PCE) reached its maximum value at 5 wt% SEBS. Table 1 summarizes the photovoltaic parameters of the devices as a function of SEBS content based on measurements from ten independently fabricated devices. Figure S1 shows the current density–voltage (*J*–*V*) characteristics of devices with 0 and 5 wt% SEBS, where the device incorporating 5 wt% SEBS exhibited a significantly enhanced FF, which served as the primary factor governing the PCE enhancement. The higher FF suggests reduced losses during charge transport and collection processes, which is further supported by the detailed analyses in the following sections. When the SEBS content exceeded 5 wt%, all parameters decreased, with a drastic decline observed at 20 wt%. This tendency was consistent with previous reports and can be attributed to the insulating nature of SEBS; excessive SEBS incorporation interrupts charge transport pathways and compromises the percolated donor-acceptor network of the BHJ layer [23,24,31]. These results suggest that an optimal amount of SEBS effectively tailors the nanoscale morphology of the BHJ layer through its role as a soft elastomeric matrix, leading to improved charge transport and reduced recombination losses, whereas excessive incorporation adversely affects the device performance. This composition-dependent behavior further indicates that the morphology-stabilizing function of an insulating soft matrix component should be balanced with the electronic percolation requirements of the BHJ system. The effects of SEBS on the morphology and charge transport process are discussed in detail in the following sections.

**Figure 2:**
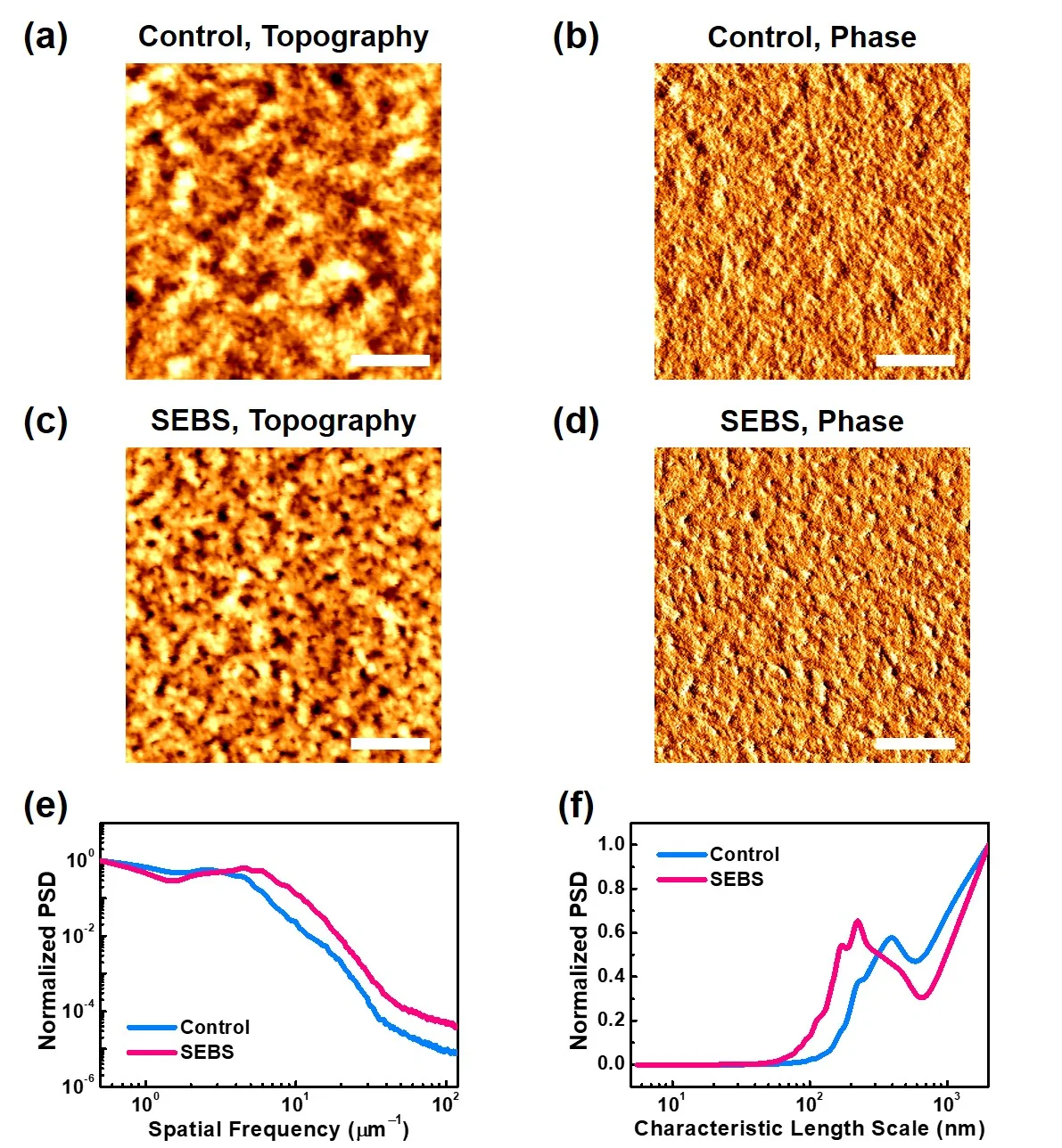
(**a**–**d**) AFM topography and phase images of the control and SEBS blend films. Scale bars indicate 500 nm. (**e**) Normalized radially averaged PSD profiles, derived from the corresponding AFM topography images, as a function of the spatial frequency. (**f**) PSD profiles plotted as a function of the corresponding lateral characteristic length scale.

To investigate the influence of SEBS incorporation on the nanoscale morphology, the surface morphologies of PM6:Y6 blend films with and without 5 wt% SEBS were examined using atomic force microscopy (AFM). Figure 2a–d present the AFM topography and phase images of the control (0 wt% SEBS) and SEBS (5 wt% SEBS) films over a 2 μm × 2 μm area. Additional magnified AFM images are provided in Figure S2 for clearer visualization of the surface morphology. Although the root-mean-square roughness slightly increased from 1.12 to 1.48 nm upon the incorporation of 5 wt% SEBS, both films exhibited smooth surfaces with low roughness, indicating that a small amount of SEBS can be incorporated as a soft polymeric matrix component without inducing severe phase separation or disrupting film formation. Interestingly, differences in the lateral morphological features were observed between the two films. Compared with the control film, the SEBS-containing film exhibited structural variations distributed over relatively smaller length scales. This tendency was also observed in the phase images, suggesting that SEBS incorporation modifies the lateral organization of the donor–acceptor network without inducing significant large-scale phase segregation.

To quantitatively analyze these lateral morphological differences, the radial power spectral density (PSD) was calculated from the AFM topography images [32,33]. Figure 2e shows the normalized PSD profiles of the blend films. Neither film exhibited significant low-frequency dominance associated with severe large-scale phase separation, indicating that the overall BHJ morphology remained relatively uniform. A distinct difference was observed in the low-to-intermediate spatial frequency region, where the SEBS film exhibited a greater spectral contribution compared with that of the control film. This behavior indicates a redistribution of morphological features toward higher spatial frequencies, corresponding to relatively smaller lateral length scales [32,33]. To further quantify this tendency, the PSD data were converted into characteristic length-scale distributions (Figure 2f). The control film was dominated by contributions from larger structures extending into the submicrometer regime, whereas the SEBS film exhibited a reduced characteristic length scale. Based on these findings, it is plausible that SEBS influences the phase-separation kinetics of the PM6:Y6 blend through partial interactions with the blend components and its role as a compliant thermoplastic elastomeric matrix. In particular, SEBS is expected to influence the diffusion and crystallization behavior of the BHJ components, regulate domain growth, and increase the relative contribution of smaller nanoscale morphological features [7,24,34]. Because excessively large domains disrupt continuous charge transport pathways and increase charge recombination in BHJ films [35,36], SEBS-mediated morphology modification can contribute to more continuous donor and acceptor networks. Therefore, the AFM and PSD results suggest that SEBS incorporation modifies the nanoscale organization of the PM6:Y6 blend toward relatively smaller characteristic morphological features. From the viewpoint of gel-related soft material design, these results indicate that an elastomeric matrix component can regulate the nanoscale organization of a functional energy-conversion layer without causing macroscopic phase instability. Nevertheless, because AFM provides information primarily on the film surface, complementary bulk-sensitive analyses such as grazing-incidence wide-angle X-ray scattering would be beneficial for further elucidating the three-dimensional BHJ morphology.

**Figure 3:**
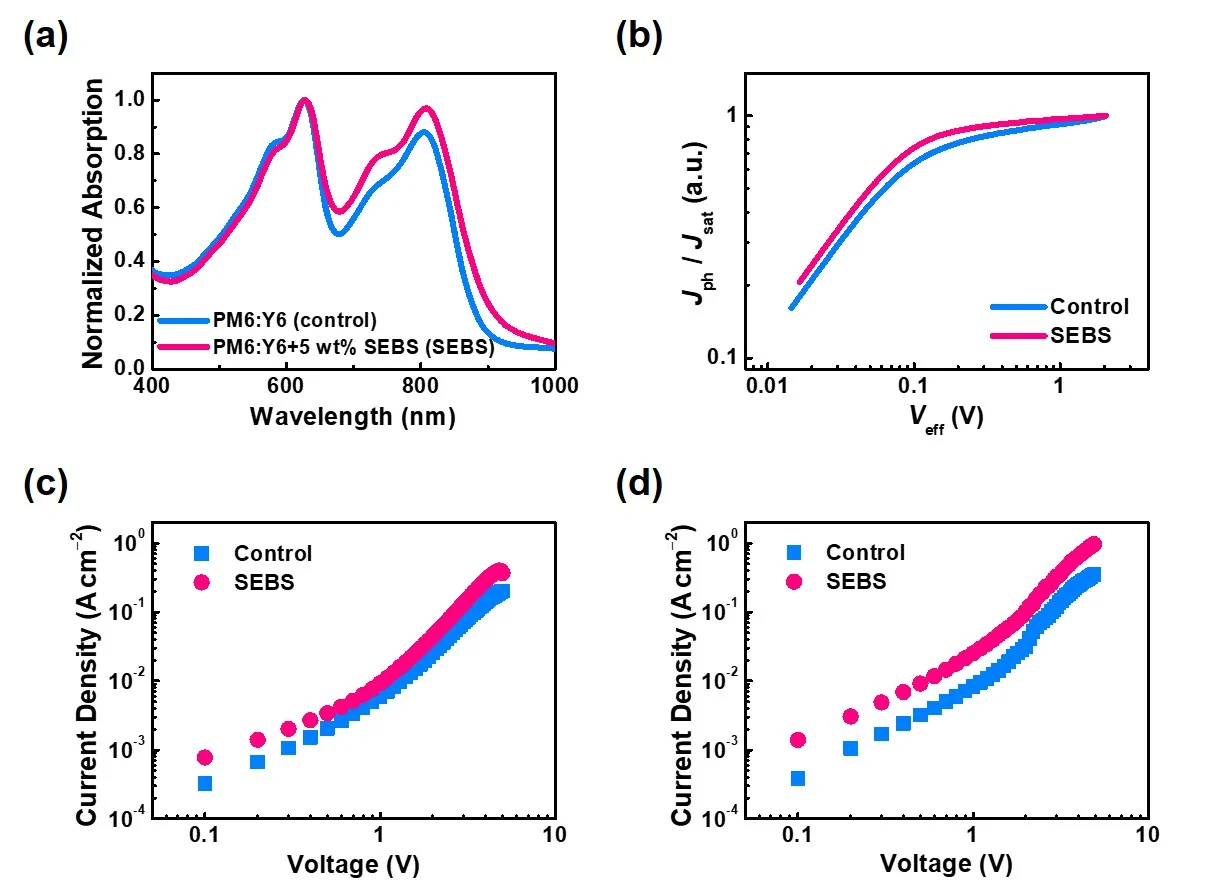
(**a**) UV–Vis absorption spectra of the control and SEBS blend films. (**b**) *J*_ph_/*J*_sat_ ratio as a function of the *V*_eff_ for the control and SEBS OPVs. Dark *J–V* characteristics of the (**c**) hole- and (**d**) electron-only devices used to determine the charge carrier mobilities based on the SCLC model.

Figure 3a presents the ultraviolet–visible (UV–Vis) absorption spectra of the control and SEBS films. Both films exhibited the characteristic absorption features of PM6 and Y6 [30], and the overall spectral profiles remained largely unchanged following the incorporation of SEBS, indicating that the additive did not disrupt the fundamental optical response of the BHJ active layer. No obvious peak shift was observed in the PM6 absorption region, which was accompanied by a marginal change in the I_(0–0)_/I_(0–1)_ ratio, suggesting that SEBS exerted only a limited influence on the molecular ordering of PM6. Interestingly, the Y6-related absorption in the SEBS film exhibited a discernible redshift along with an enhanced long-wavelength shoulder. These spectral changes suggest subtle modifications in Y6 aggregation and local packing rather than providing direct evidence of strengthened intermolecular interactions [37,38]. Such changes indicate that SEBS influences the local organization of the acceptor phase without substantially altering the overall optical response of the blend. Notably, when SEBS was introduced into the PM6:Y6 blend in the absence of 1-chloronaphthalene (CN), only marginal changes were observed in the peak positions and vibronic features. As shown in Figure S3, the UV–Vis absorption spectra of the PM6:Y6 blend films with and without 5 wt% SEBS were nearly identical in the absence of CN. This comparison indicates that the influence of SEBS on Y6 aggregation and local packing becomes more pronounced in the presence of CN. High-boiling-point CN is known to prolong the film-formation process and enable continued molecular rearrangement and aggregation of Y6 during the delayed evaporation of residual CN [34,39]. Accordingly, the present results suggest that CN primarily regulates the aggregation and phase-evolution kinetics, while SEBS modifies the local packing environment within this CN-mediated film-formation process. This interpretation accounts for the more pronounced spectral modulation observed when SEBS is incorporated in the presence of CN.

Figure 3b shows the normalized photocurrent ratio (*J*_ph_/*J*_sat_) as a function of the effective voltage (*V*_eff_) for the devices, where *J*_ph_ is the photocurrent density, defined as the difference between the current densities measured under illumination and in the dark, and *J*_sat_ is the saturation photocurrent density obtained at a sufficiently high *V*_eff_, at which the photogenerated charges are assumed to be almost completely dissociated and collected [40,41]. Accordingly, the *J*_ph_/*J*_sat_ ratio represents the fraction of photogenerated charges that are effectively extracted at a given *V*_eff_ and serves as a useful indicator for evaluating the charge dissociation and collection efficiencies in BHJ OPVs. Notably, the SEBS device exhibited consistently higher *J*_ph_/*J*_sat_ values than the control device across the entire *V*_eff_ range, with the difference becoming more pronounced in the low-to-intermediate *V*_eff_ region, which is most relevant under practical operating conditions. These results indicate that the incorporation of SEBS promotes more efficient charge dissociation and collection, which is consistent with the observed enhancement in the OPV performance. Figure 3c,d present the *J–V* characteristics of the hole- and electron-only devices, respectively, which were used to investigate the charge transport characteristics of the blend films [42]. Based on space-charge-limited current (SCLC) fitting, the hole mobility increased from 1.19 × 10^−4^ cm^2^ V^−1^ s^−1^ for the control film to 2.48 × 10^−4^ cm^2^ V^−1^ s^−1^ for the SEBS film, while the electron mobility increased from 2.13 × 10^−4^ to 3.25 × 10^−4^ cm^2^ V^−1^ s^−1^. In addition to the increase in the absolute mobilities, the transport balance was also improved, as reflected by the increase in the mobility ratio (*μ*_h_/*μ*_e_) from 0.56 to 0.76. More balanced charge transport is favorable for suppressing space-charge accumulation and reducing recombination losses [43,44]. Because SEBS is electrically insulating, the enhanced carrier mobilities are unlikely to arise from direct charge transport through the SEBS component, but rather from SEBS-induced changes in nanoscale organization and local packing that promote more effective charge transport pathways within the semiconducting PM6:Y6 network. Together with the improved transport balance and charge collection, these morphology-mediated transport characteristics provide a reasonable explanation for the enhanced photovoltaic performance of the SEBS OPV.

**Figure 4:**
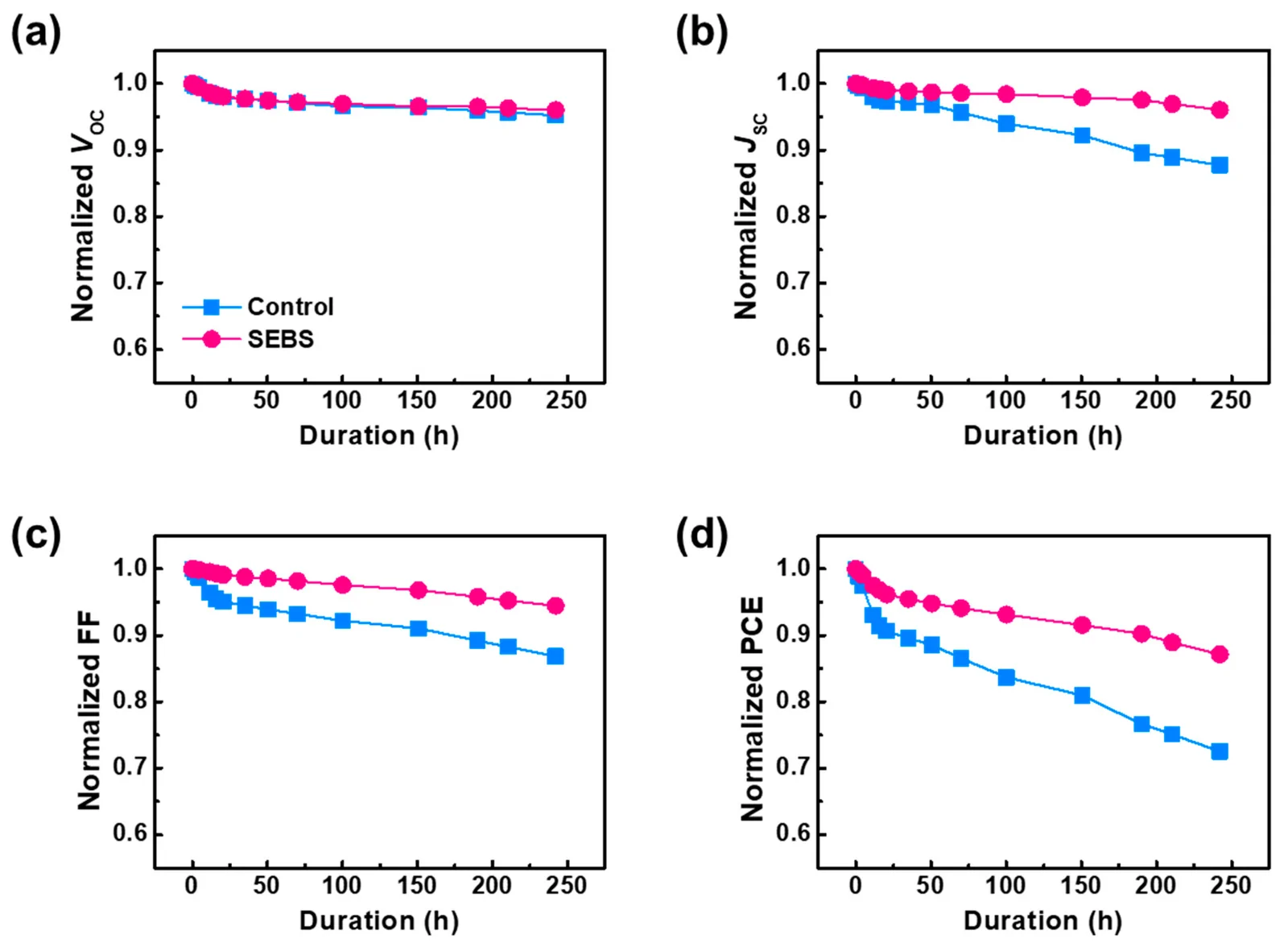
Time-dependent evolution of the normalized photovoltaic parameters of the control and SEBS OPVs during thermal aging at 85 °C: (**a**) *V*_OC_, (**b**) *J*_SC_, (**c**) FF, and (**d**) PCE.

In addition to achieving high PCEs, long-term operational stability is a critical prerequisite for the practical deployment of OPV technologies. To evaluate the effect of SEBS incorporation on device stability, the encapsulated OPVs were subjected to prolonged thermal aging under dark conditions at an elevated temperature to accelerate thermally induced morphological evolution within the BHJ active layers. Figure 4 presents the time-dependent variations in the photovoltaic parameters of the control and SEBS OPVs measured over 10 days at 85 °C. The relatively small decrease in *V*_OC_ observed for both devices indicates that changes in the built-in potential and overall energetic landscape were not the primary factors governing performance degradation during thermal aging. In contrast, distinct degradation behaviors were observed in *J*_SC_ and FF for the control and SEBS OPVs. The control device exhibited a noticeable reduction in both *J*_SC_ and FF with increasing aging time, whereas these losses were substantially mitigated in the SEBS device under the same conditions. This contrast suggests that the control active layer underwent thermally induced morphological evolution that adversely affected charge generation and extraction, most likely through progressive phase separation and domain coarsening within the BHJ active layer [7,45]. Indeed, PM6:Y6-based OPVs have been widely reported to exhibit poor thermal durability, and the degradation behavior observed in the control device in this study was consistent with this general tendency [8,28]. By contrast, the SEBS device exhibited significantly suppressed losses in both *J*_SC_ and FF, indicating that SEBS acts as a compliant elastomeric matrix that mitigates thermally induced morphological changes in the active layer, thereby suppressing excessive domain growth and preserving a more favorable BHJ structure for efficient charge transport and collection. Notably, the control device dropped below the T_80_ level, defined as 80% retention of the initial PCE, after approximately 160 h, whereas the SEBS device remained above T_80_ even after 10 days of continuous thermal aging. These results demonstrate that the soft polymeric matrix-mediated stabilization induced by SEBS is not limited to initial morphology regulation but also contributes to the long-term retention of functional charge-transport pathways in non-gel BHJ active layers.

**Figure 5:**
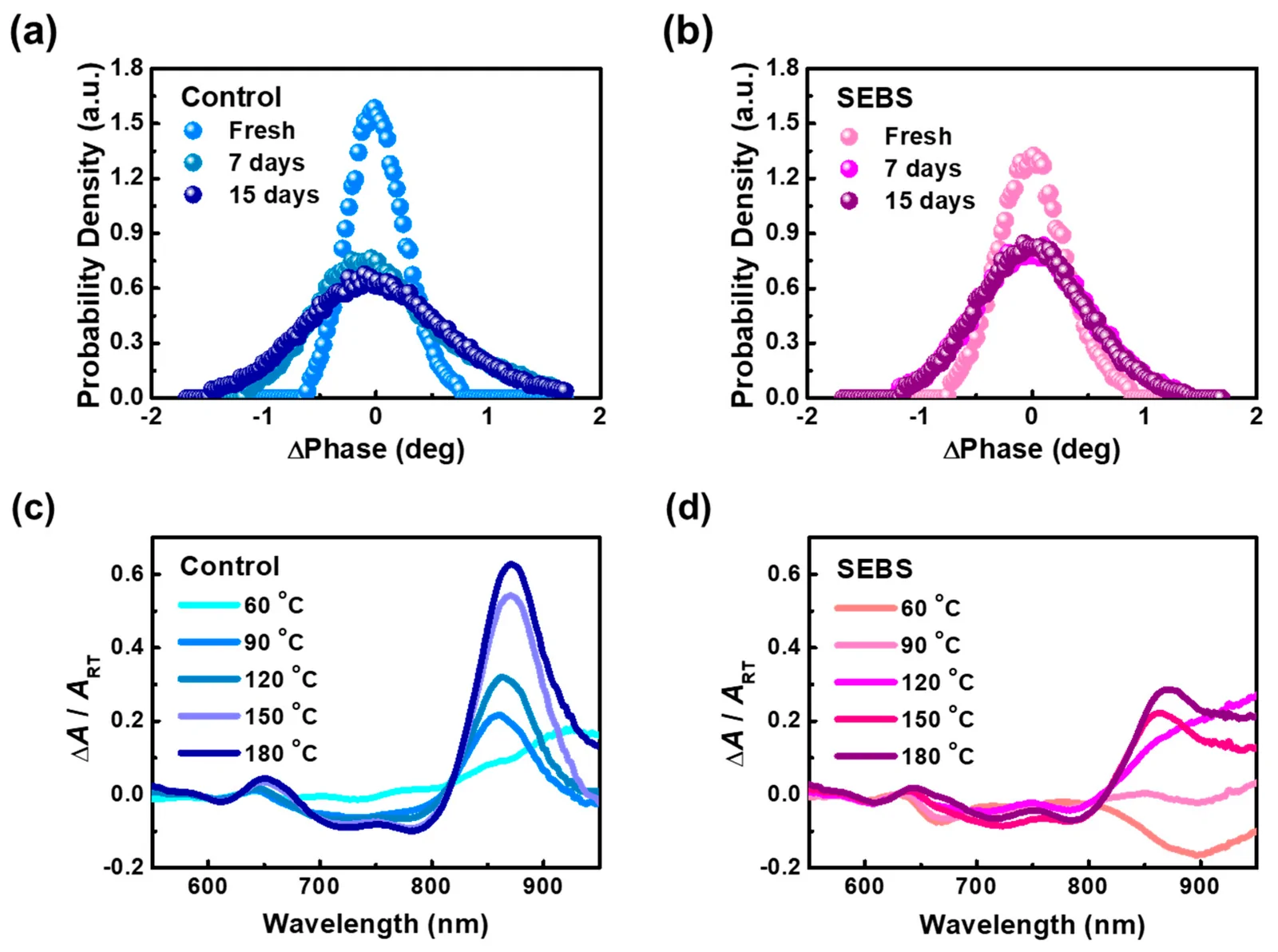
Phase-angle distributions derived from the AFM phase images of the (**a**) control and (**b**) SEBS blend films as a function of the thermal aging duration at 85 °C. Relative changes in the UV–Vis absorbance spectra of the (**c**) control and (**d**) SEBS blend films after thermal annealing at different temperatures. The relative absorbance change was calculated with respect to the spectrum measured at room temperature.

To elucidate the mechanism underlying the improved thermal durability of the SEBS-containing BHJ layer, the thermally induced morphological evolution of the BHJ active layer films was investigated. The AFM aging intervals were selected to provide complementary structural snapshots relative to the 10-day device-aging experiment. The 7-day condition (168 h) represents an intermediate stage close to the T_80_ crossover of the control device, whereas the 15-day condition (360 h) extends the morphological evaluation beyond the device-aging period to assess whether the SEBS-induced stabilization persists under prolonged thermal stress. Figure 5a,b show the histograms of the AFM phase signals extracted from the phase images obtained after different thermal treatment durations at 85 °C. A phase histogram statistically represents the distribution of phase values from all pixels in an AFM phase image, reflecting the distribution of the local AFM phase contrast near the film surface [46]. A narrow and sharp histogram indicates minimal spatial variation in the phase signal, whereas histogram broadening accompanied by a decrease in peak density indicates that the phase signals are distributed over a wider range. For the control film, the fresh sample exhibited a narrow and sharply centered histogram at approximately Δphase = 0, indicating a uniform local phase contrast in the initial BHJ morphology. After thermal aging at 85 °C for 7 days, the peak density decreased and the histogram broadened relative to that of the fresh film. This trend became more pronounced after additional thermal treatment (15 days), with the distribution tail extending over a wider Δphase range. These results demonstrate that the spatial variation in the AFM phase contrast increased significantly during thermal aging in the control film. In contrast, the SEBS film exhibited much smaller changes in the phase histogram under identical thermal conditions. The fresh SEBS film also showed a narrow phase-signal distribution centered near Δphase = 0, and although some broadening was observed after thermal aging, the increase in histogram width was considerably suppressed compared with that of the control film. Interestingly, the overall histogram profiles after 7 and 15 days of aging remained nearly identical, implying that further changes in the local phase contrast were largely restrained during prolonged thermal aging. These morphological trends are consistent with the device-aging results, in which the control OPV exhibited pronounced performance degradation near 160 h, whereas the SEBS device maintained substantially higher performance throughout the 240 h aging period. Figure S4 shows the evolution of the phase and topography *R_q_* values during thermal treatment at 85 °C for 15 days. The increase in the phase *R_q_* was substantially smaller for the SEBS film under thermal stress, which was in good agreement with the phase histogram results. Furthermore, compared with the control film, the roughness of the SEBS film increased only moderately after aging and remained relatively stable even after prolonged thermal treatment. These results indicate that SEBS mitigates thermally induced morphological evolution in the BHJ active layer, including changes in the local phase contrast, surface roughening, and large-scale aggregation during prolonged thermal aging. This suppression is attributed to the compliant SEBS matrix, which limits thermally activated molecular rearrangement and helps maintain a more spatially uniform morphology under thermal stress.

To gain further insight into the suppressed morphological evolution of the SEBS-containing films, temperature-dependent UV–Vis absorption spectra were measured following stepwise thermal treatment. Figure S5 shows the UV–Vis absorption spectra of the control and SEBS films after stepwise heating from room temperature (RT) to 180 °C. To directly compare the extent of the thermally induced spectral evolution, the relative absorbance change was calculated with respect to the room-temperature spectrum using the following relationship: (A(λ,T) − A(λ,RT))/A(λ,RT) (Figure 5c,d). For the control film, the relative absorbance change became increasingly pronounced with increasing temperature, particularly in the long-wavelength absorption region associated with Y6-dominated molecular aggregation and packing [37]. The substantial positive change in this region indicates that thermal treatment significantly modified the low-energy absorption feature of the blend film, suggesting thermally induced aggregation of the acceptor-rich domains [8]. This spectral evolution is consistent with the AFM results, in which the control film exhibited a pronounced broadening of the phase-signal distribution and increased surface roughness after thermal aging. In contrast, the SEBS film exhibited much smaller relative spectral changes in the long-wavelength region under the same thermal treatment conditions, indicating that SEBS, acting as a compliant elastomeric matrix, effectively mitigated excessive changes in the aggregation and packing state of the Y6-rich phase. Consequently, SEBS limited thermally driven molecular rearrangement and suppressed uncontrolled morphological evolution, supporting a matrix-restricted mechanism for stabilizing the BHJ morphology under thermal stress.

The combined experimental results provide compelling evidence that the control PM6:Y6 blend undergoes thermally induced morphological evolution, whereas the SEBS-containing blend maintains a more stable nanoscale organization under thermal stress. In the control film, uncontrolled morphological evolution driven by thermal treatment, likely associated with excessive molecular diffusion and domain growth, disrupted the initially optimized BHJ nanostructure and destabilized the charge generation and collection pathways. This interpretation is consistent with the substantial decline in photovoltaic performance observed for the thermally aged control devices. In contrast, the incorporation of SEBS effectively mitigated thermally induced morphological changes by suppressing excessive coarsening and aggregation, thereby preserving the initial BHJ morphology during prolonged aging. Figure S6 provides a schematic illustration of the proposed SEBS-mediated morphology stabilization mechanism during prolonged thermal aging. This morphology-stabilizing effect contributes to the retention of efficient charge transport and collection pathways, providing a reasonable explanation for the improved PCE retention of the SEBS OPVs under thermal stress. These results demonstrate that, in addition to its previously established role as a mechanical modifier for improving the flexibility of OPV active layers [24,25,26,27], SEBS serves as an effective soft elastomeric matrix component that stabilizes functional BHJ active layers under thermal stress. From the viewpoint of gel-related soft material design, the present results indicate that compliant polymeric matrix components can be used to regulate molecular mobility, suppress thermally induced morphological evolution, and preserve functional charge-transport pathways in energy-conversion layers. Therefore, SEBS-mediated soft polymeric matrix stabilization provides a practical route for simultaneously enhancing the initial device performance and long-term thermal stability of OPVs. The integration of SEBS-mediated morphological stabilization with complementary thermal-management strategies could further contribute to the development of efficient and thermally durable photovoltaic systems [47,48].

## 3. Conclusions

In summary, we demonstrated that incorporating a small amount of the thermoplastic elastomer SEBS provides an effective soft polymeric matrix-mediated strategy for simultaneously improving the efficiency and thermal stability of OPVs. The incorporation of 5 wt% SEBS into the PM6:Y6 blend increased the PCE from 14.27% to 15.22%, mainly because of enhanced hole and electron mobilities, more balanced charge transport, and more efficient charge collection. AFM and PSD analyses revealed that SEBS regulated the nanoscale organization of the BHJ blend while maintaining a smooth surface without inducing severe aggregation or phase separation. UV–Vis absorption analysis further revealed subtle changes in the aggregation and local packing characteristics of Y6 when SEBS was combined with CN, indicating that its morphological effect is closely associated with additive-mediated aggregation and phase evolution during film formation. More importantly, the SEBS OPVs exhibited substantially reduced performance degradation compared with the control devices during prolonged thermal aging at 85 °C. The enhanced thermal robustness is attributed to the ability of the compliant elastomeric SEBS matrix to suppress thermally driven morphological evolution, including excessive phase separation and domain coarsening, thereby preserving efficient charge transport pathways. Collectively, these results demonstrate the important role of SEBS as a soft elastomeric matrix component in regulating the initial nanoscale morphology and maintaining an optimized BHJ nanostructure under thermal stress. From the viewpoint of gel-related soft material design, this study demonstrates that an elastomeric matrix component can stabilize functional BHJ energy-conversion layers by regulating molecular rearrangement, suppressing thermally induced morphological evolution, and preserving charge-transport pathways. These findings highlight soft polymeric matrix stabilization based on gel-related soft material concepts as a simple and promising approach for overcoming the efficiency–stability bottleneck and realizing thermally robust OPVs.

## 4. Materials and Methods

### 4.1. Device Fabrication

The ITO-coated glass substrates were sequentially cleaned by ultrasonication in deionized water, acetone, and isopropyl alcohol for 15 min each. After drying, the substrates were treated with UV-ozone for 15 min. A ZnO electron transport layer was formed by spin-coating a ZnO precursor solution onto the ITO substrates. The precursor solution was prepared by dissolving zinc acetate dihydrate (Sigma-Aldrich, St. Louis, MO, USA) and ethanolamine (Sigma-Aldrich, St. Louis, MO, USA) in 2-methoxyethanol (Sigma-Aldrich, St. Louis, MO, USA) [49]. The resulting ZnO films were thermally annealed in air at 300 °C for 10 min. The substrates were then transferred to a nitrogen-filled glove box, where the active layer solution was spin-coated at 3000 rpm for 30 s. The control active layer solution was prepared by dissolving PM6 (Ossila, Sheffield, England) and Y6 (Ossila, Sheffield, England) in chloroform (Sigma-Aldrich, St. Louis, MO, USA) at a weight ratio of 1:1, with 0.5 vol% CN (Sigma-Aldrich, St. Louis, MO, USA) used as a solvent additive. For the SEBS blend solutions, SEBS (Sigma-Aldrich) was added at 5, 10, and 20 wt% relative to the total solid content while maintaining the PM6:Y6 weight ratio at 1:1. The total solid concentration, including PM6, Y6, and SEBS, was fixed at 16 mg mL^−1^ for all compositions. The active layer thicknesses of the films containing 0, 5, 10, and 20 wt% SEBS were maintained at approximately 100 nm with variations of approximately ±5 nm. Following active layer deposition, MoO_3_/Ag electrodes with thicknesses of 10 and 100 nm were deposited by thermal evaporation under high vacuum (<10^−7^ Torr). The completed devices were encapsulated using glass covers and UV-curable epoxy.

### 4.2. Characterization

The *J*–*V* characteristics of the OPV devices were measured using a Keithley 2450 source measure unit under simulated AM 1.5G illumination at an intensity of 100 mW cm^−2^. Illumination was provided by a solar simulator (SimuLight SS-LED50S, McScience, Suwon, Republic of Korea), and the light intensity was calibrated using a certified Si reference cell (S301-K125, McScience, Suwon, Republic of Korea). The FF was calculated as FF = (*V*_MPP_ ∙ *J*_MPP_)/(*V*_OC_ ∙ *J*_SC_), where *V*_MPP_ and *J*_MPP_ are the voltage and current density at the maximum power point, respectively. The optical absorption spectra of the active layer films were recorded using a UV–Vis spectrophotometer (OPTIZEN POP, KLAB, Daejeon, Republic of Korea). Temperature-dependent absorption measurements were performed by annealing the active layer films at selected temperatures for 30 min, followed by spectral acquisition after each annealing step. The annealing temperature was increased from RT to 180 °C in 30 °C increments. Charge carrier mobilities were determined using the SCLC method. Hole-only devices were fabricated with the structure ITO/poly(3,4-ethylenedioxythiophene):poly(styrenesulfonate)/active layer/MoO_3_/Ag, while electron-only devices were fabricated with the structure ITO/ZnO/active layer/2,9-bis [3-(dimethyloxidoamino)propyl]anthra [2,1,9-def:6,5,10-d′e′f′]diisoquinoline-1,3,8,10(2H,9H)-tetrone/Ag. The dark *J–V* characteristics of these single-carrier devices were measured, and the carrier mobilities were extracted using the Mott–Gurney relation: $J=\frac{9}{8}\varepsilon_{0}\varepsilon_{r}\mu \frac{V^{2}}{L^{3}}$, where *J* is the current density, *ε*_0_ is the vacuum permittivity, *ε*_r_ is the relative dielectric constant, *μ* is the carrier mobility, *V* is the applied voltage used for the fitting, and *L* is the active layer thickness [42]. The active layer thickness was approximately 100 nm, and *ε*_r_ = 3 was used for the mobility calculations. The reported carrier mobilities were obtained from four independently fabricated devices for each condition. The fitting regions were identified from the log *J*–log *V* plots where the slopes approached 2, indicating an approximately quadratic dependence of current density on voltage as described by the Mott–Gurney relation. Linear fits of *J* versus *V^2^* yielded R^2^ values of 0.9979 and 0.9972 for the control and SEBS hole-only devices, respectively, and 0.9895 and 0.9954 for the control and SEBS electron-only devices, respectively. AFM (XE-150, Park Systems, Gwacheon, Republic of Korea) was used to characterize the surface morphology and phase contrast of the active layer films in tapping mode. PSD analysis was performed by applying a two-dimensional fast Fourier transform to the height images. The resulting two-dimensional PSD maps were radially averaged to obtain one-dimensional PSD profiles as a function of spatial frequency. For phase analysis, all phase images were processed under identical analysis conditions, and the phase-value distributions were extracted as histograms to compare the degree of local phase-contrast variation. Thermal stability tests were conducted using encapsulated devices. The devices were aged on a preheated digital hot plate at 85 °C in the dark under ambient atmosphere. The photovoltaic parameters were periodically measured at RT under simulated AM 1.5G illumination.

## Figures and Tables

**Table 1:** Photovoltaic parameters of the OPVs as a function of SEBS content under AM 1.5G illumination at 100 mW cm^−2^. The error ranges represent standard deviations obtained from ten independently fabricated devices.

SEBS Content (wt%)	*V*_OC_ (V)	*J*_SC_ (mA cm^−2^)	FF (%)	PCE (%)
0	0.85 ± 0.01	24.96 ± 0.16	67.27 ± 1.09	14.27 ± 0.19
5	0.86 ± 0.01	25.03 ± 0.21	70.91 ± 1.07	15.22 ± 0.21
10	0.85 ± 0.01	24.83 ± 0.18	65.69 ± 1.10	13.93 ± 0.15
20	0.85 ± 0.01	22.14 ± 0.14	57.42 ± 0.93	10.83 ± 0.11

## Data Availability

Data is contained within the article or Supplementary Material.

## Supplementary Materials

The following supporting information can be downloaded at: https://www.mdpi.com/article/10.3390/gels12080750/s1. Figure S1: *J–V* characteristics of the control and SEBS-based OPVs; Figure S2: Magnified AFM topography and phase images of the control and SEBS-containing blend films. Scale bars indicate 200 nm; Figure S3: UV-Vis absorption spectra of PM6:Y6 blend films with and without 5 wt% SEBS in the absence of CN; Figure S4: Evolution of the (a) phase and (b) topography *R_q_* values of the control and SEBS blend films during thermal treatment at 85 °C for 15 days; Figure S5: UV-Vis absorption spectra of the control and SEBS blend films after stepwise heating from RT to 180 °C; Figure S6: Schematic illustration of SEBS-mediated BHJ morphology stabilization under thermal stress. The control PM6:Y6 blend undergoes thermally induced aggregation and domain coarsening during aging, leading to disruption of the donor–acceptor network. In the SEBS-containing blend, the compliant thermoplastic elastomer matrix mitigates excessive molecular rearrangement and preserves the nanoscale BHJ morphology, resulting in enhanced thermal robustness of the OPVs.
