# Supplementary material for: Soft Polymeric Matrix-Mediated Stabilization of Bulk Heterojunction Morphology for Thermally Robust Organic Photovoltaics
Source: Gels. 2026 Aug 21;12(8):750. doi: 10.3390/gels12080750 (PMC13512396; doi:10.3390/gels12080750)
Supplement: Supplementary file 1 [file gels-12-00750-s001.zip › gels-4493921-supplementary.pdf]

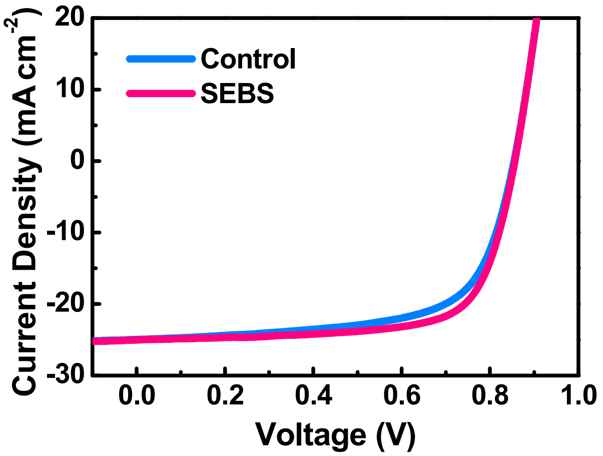


**Figure S1.** *J–V* characteristics of the control and SEBS-based OPVs.

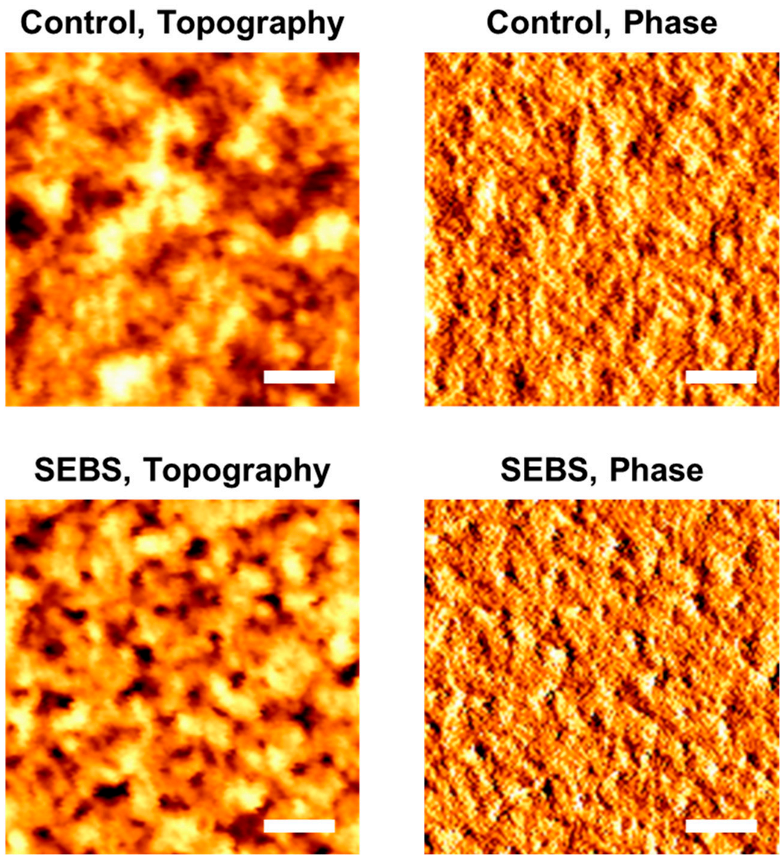


**Figure S2.** Magnified AFM topography and phase images of the control and SEBS-containing blend films. Scale bars indicate 200 nm.

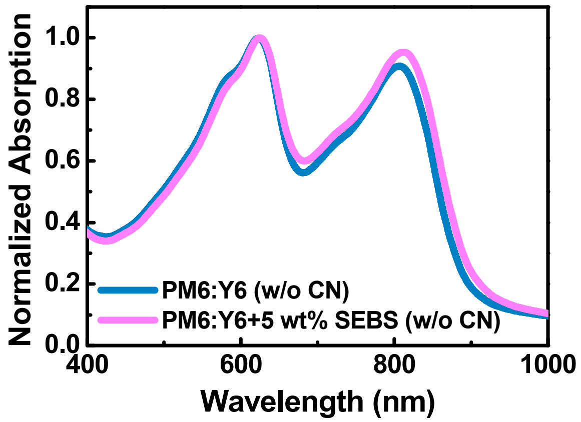


**Figure S3.** UV-Vis absorption spectra of PM6:Y6 blend films with and without 5 wt% SEBS in the absence of CN.

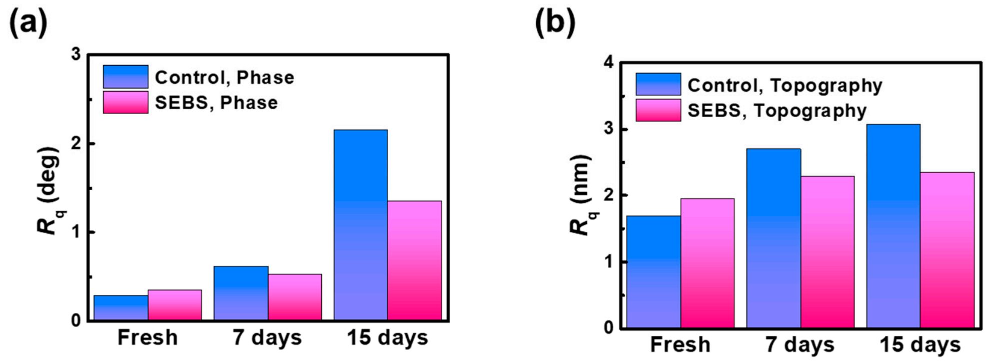


**Figure S4.** Evolution of the (a) phase and (b) topography $R_q$ values of the control and SEBS blend films during thermal treatment at 85 °C for 15 days.

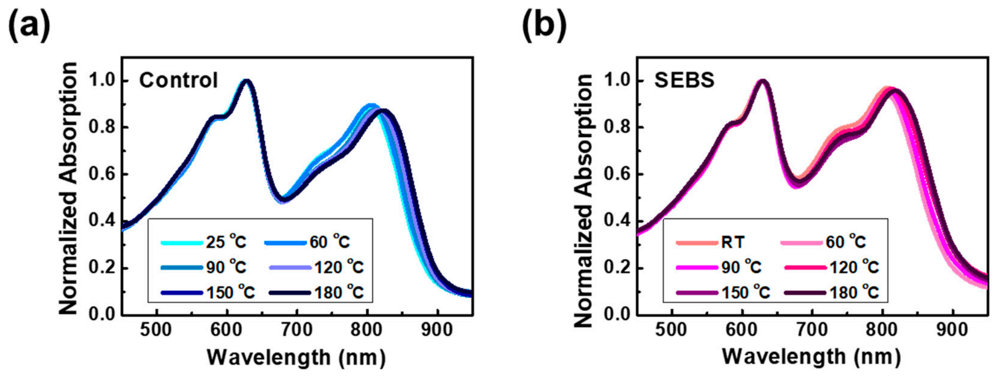


**Figure S5.** UV-Vis absorption spectra of the control and SEBS blend films after stepwise heating from RT to 180 °C.

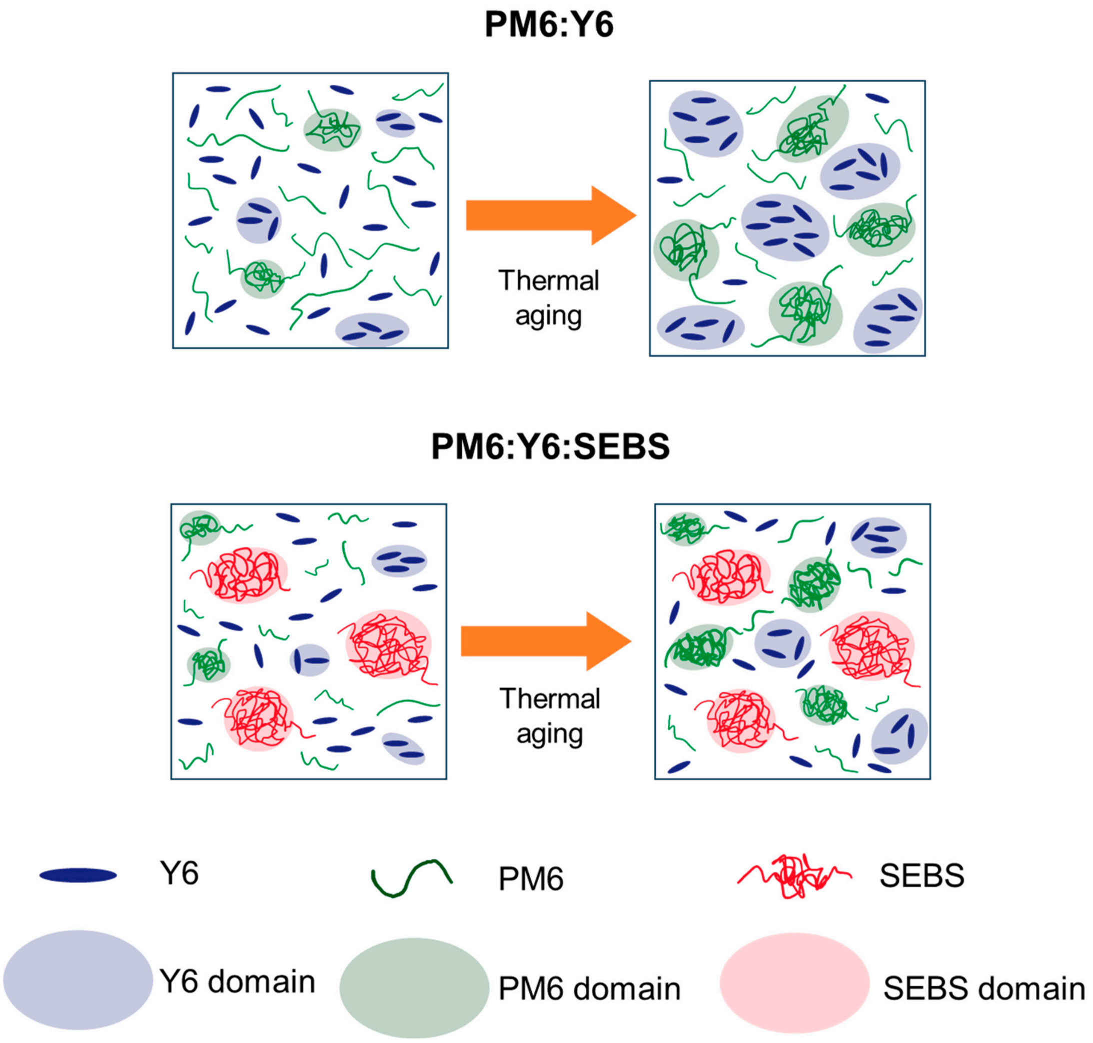


**Figure S6.** Schematic illustration of SEBS-mediated BHJ morphology stabilization under thermal stress. The control PM6:Y6 blend undergoes thermally induced aggregation and domain coarsening during aging, leading to disruption of the donor–acceptor network. In the SEBS-containing blend, the compliant thermoplastic elastomer matrix mitigates excessive molecular rearrangement and preserves the nanoscale BHJ morphology, resulting in enhanced thermal robustness of the OPVs.
